# Additive-manufactured ceramics for dental restorations: a systematic review on mechanical perspective

**DOI:** 10.3389/fdmed.2025.1512887

**Published:** 2025-02-10

**Authors:** Yuqing Lu, Anouk van Steenoven, Amanda Maria de Oliveira Dal Piva, João Paulo Mendes Tribst, Li Wang, Cornelis J. Kleverlaan, Albert J. Feilzer

**Affiliations:** ^1^ Department of Dental Materials Science, Academic Centre for Dentistry Amsterdam (ACTA), Universiteit van Amsterdam and Vrije Universiteit, Amsterdam, Netherlands; ^2^ Department of Reconstructive Oral Care, Academic Centre for Dentistry Amsterdam (ACTA), Universiteit van Amsterdam and Vrije Universiteit, Amsterdam, Netherlands; ^3^ Jiangsu Key Laboratory of Advanced Food Manufacturing Equipment and Technology, School of Mechanical Engineering, Jiangnan University, Wuxi, Jiangsu, China; ^4^ Institute of Advanced Technology, Jiangnan University, Wuxi, Jiangsu, China

**Keywords:** additive manufacturing, prosthodontics, dental restoration, ceramics, strength, vat photopolymerization

## Abstract

**Background:**

Additive manufacturing (AM) is rapidly expanding as a substitute for conventional heat-pressing and milling techniques for ceramic restorations. However, experimental and clinical evidence on the mechanical properties and performance of the final ceramic products is yet insufficient. This systematic review aimed to update the latest advances in additive manufacturing of restorative ceramics with a focus on their mechanical properties.

**Methods:**

This systematic review was structured using the 5-step methodology based on the research question: what are the mechanical properties of additive-manufactured restorative ceramics in comparison with subtractive manufacturing? The electronic literature search was performed independently by 2 authors in the following databases: PubMed/MEDLINE, Web of Science, and Scopus. Published articles from 2019 to 2023 were screened, analysed and the relevant papers were selected for inclusion in this review.

**Results:**

A total of 40 studies were included. The available ceramics include zirconia, alumina and alumina-zirconia composites, lithium disilicate, porcelain and fluorapatite glass ceramic. The mechanical properties were summarized according to material and technique: density (15 studies), flexural strength (31 studies), fracture toughness (7 studies), Young's modulus (7 studies), hardness (11 studies) and performance (7 studies). Overall, the properties exhibited an upward trend toward the values of conventional techniques. Typical processing defects, including porosity, agglomerates, cracks, surface roughness, and other defects, were also analyzed.

**Conclusions:**

With significant technological advancements, the mechanical properties of AM ceramics have come close to ceramics by conventional manufacturing, whereas their reliability, the influence of printing layer orientations, and long-term performance still need further investigation.

## 1 Introduction

**Figure 1:**
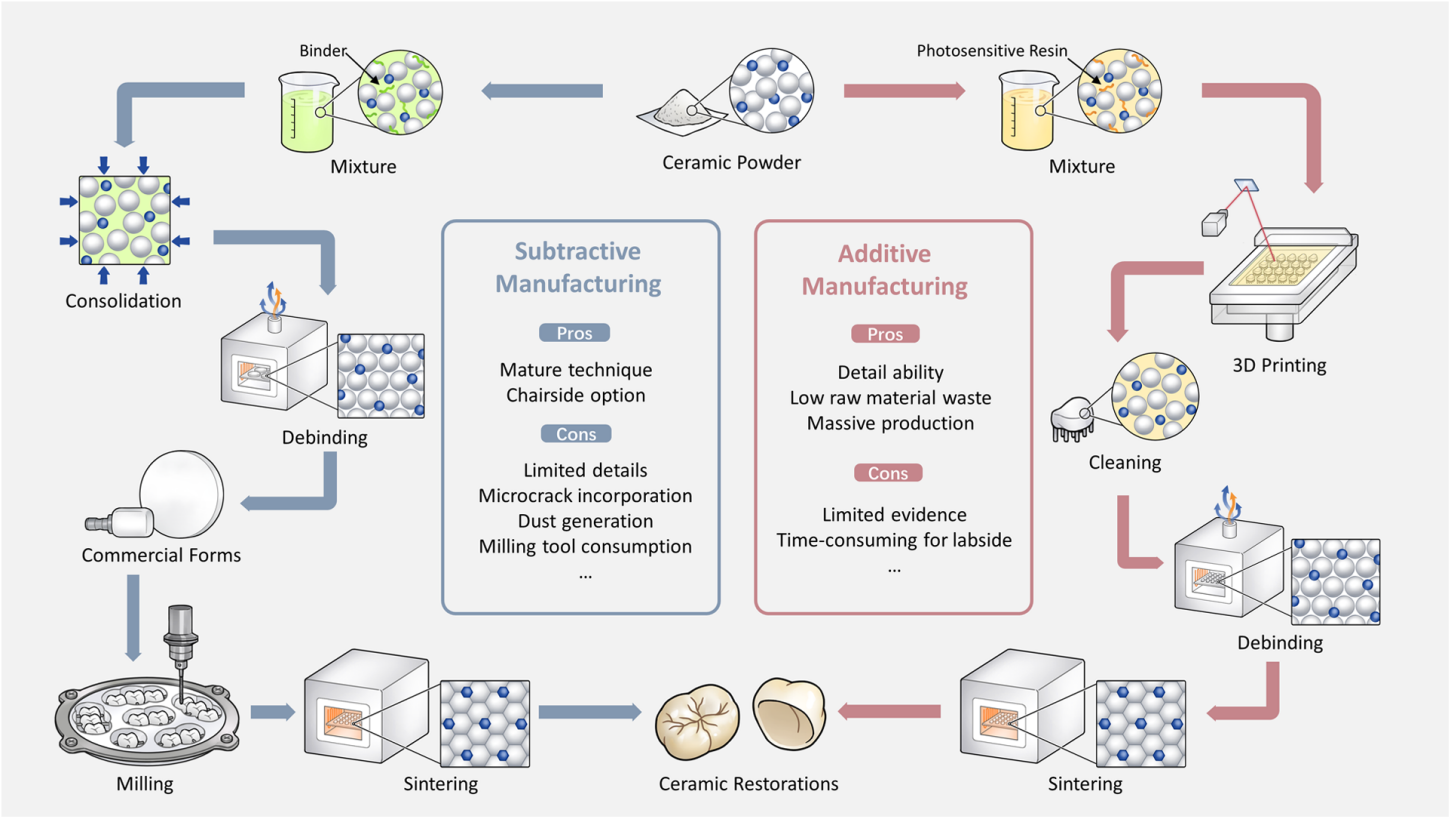
Comparison between SM and AM techniques for zirconia restorations.

Ceramic materials play an irreplaceable role in prosthetic dentistry due to their superior mechanical properties, biocompatibility, chemical stability and aesthetic appearance. Dental ceramics such as polycrystalline and glass ceramics are suitable for permanent restorations such as veneers, inlays/onlays, crowns, fixed partial dentures and implant suprastructures (1–3). To shape ceramic restorations, subtractive manufacturing (SM), also known as milling, is widely utilized. In this technique, the desired geometry is obtained by a milling machine that works in specific paths from a ceramic blank (Figure 1). The blank, usually in the shape of a block or disc, can be either fully or partially sintered/crystallized, resulting in hard or soft machining. For that, dental restorations are digitally designed and as three-dimensional (3D) files imported into the milling unit to be manufactured, either chairside or labside. Despite its advantages, SM is burdened with several drawbacks: its capacity to reproduce intricate geometry is limited due to constraints posed by milling tools' diameter, length, and machine axes (4, 5); it generates substantial raw material wastage, contributing to pollution and complicating dust recycling efforts (6); microcracks may form during milling, potentially compromising the restorations' mechanical integrity (7, 8); moreover, the cutting tool experiences frequent wear, necessitating regular replacements (4, 5); finally, for mass production, the technique's efficiency is hampered by the limitation of milling only one restoration at a time when using a block.

Additive manufacturing (AM), namely 3D printing or rapid prototyping, has rapidly evolved as a substitute for conventional techniques with higher capacity to manufacture complex and detailed geometries (5, 9–11). AM enables the production of items made of nearly all types of materials including metals, polymers, and ceramics (5). AM offers several inherent advantages over SM, including enhanced surface detailing, suitability for mass production, reduction of material waste, etc. (12). However, despite the wide application of dental ceramics in restorative dentistry, within the authors' knowledge, the clinical usage of AM ceramics is extremely limited compared to metals and polymers (5). This can be associated with the challenges in technique development and license authorization, leading to few available printable dental ceramic materials on the market. One of the key concerns is the mechanical aspect (5, 13, 14). The indications of ceramic products are strictly determined by their initial mechanical properties according to ISO 6,872 (15), while the restorations' lifetime depends on their resistance to humidity, fatigue as well as occlusal wear in the oral environment. Experimental and clinical evidence on the mechanical properties of AM materials and the long-term performance of printed restorations is yet insufficient.

Therefore, this review was conducted to update the status and address the challenges of AM dental ceramics, with a focus on their mechanical properties. This review focused on two main categories of dental restorative ceramics: polycrystalline and glass ceramics. Although ceramic-resin composites also contain a ceramic component, their processing techniques and properties are unique, leading to clinical indications and evaluation approaches that differ considerably from ceramic materials. Therefore, ceramic-resin composites were not included in the scope of this paper.

## 2 Methods

**Figure 2:**
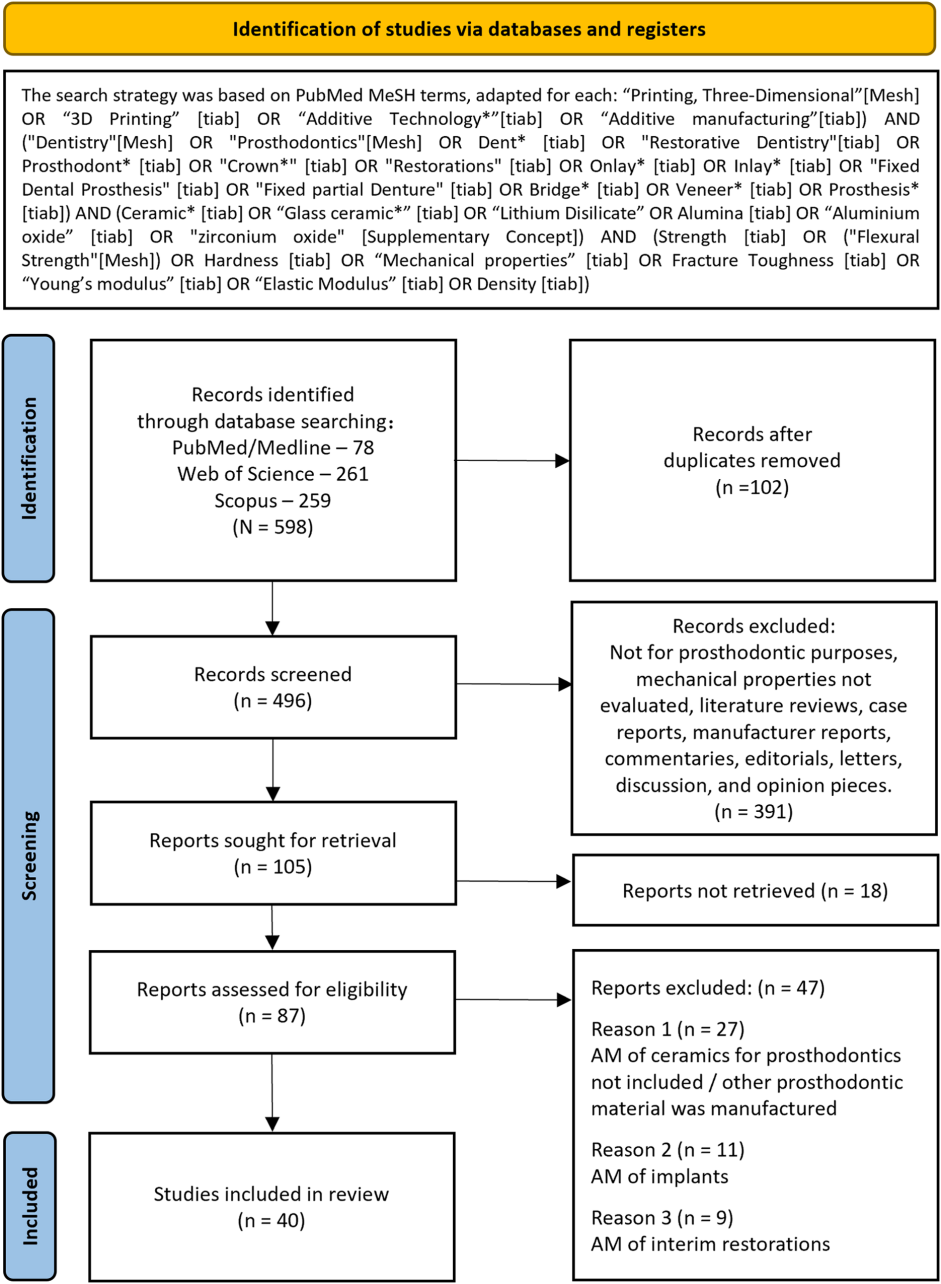
PRISMA-ScR flow diagram showing selection of articles for this review.

This review was structured based on the 5-step methodology proposed by Arksey and O'Malley (16): Identifying the research question; detecting relevant studies; study selection; charting the data; and gathering, summarizing, and reporting results. The research question consisted of “What are the mechanical properties of additive-manufactured restorative ceramics in comparison with subtractive manufacturing?” The search strategy in Figure 2 was adapted for each electronic database (PubMed/MEDLINE, Web of Science, and Scopus) and performed independently by two authors.

The inclusion criteria were studies published from 2019 to 2023 in English and studies examining AM of dental ceramics. The examined parameters include density and mechanical properties such as flexural strength, fracture toughness, Young's modulus and hardness. These studies included clinical trials, randomized controlled trials, or experimental *in vitro* studies. The exclusion criteria were studies not meeting the inclusion criteria, studies on AM ceramics that were not for prosthodontic purposes, or literature reviews, case reports, manufacturer reports, protocol optimization, or commentaries, editorials, letters, discussion, opinion pieces and unavailability of full text. The details of the search strategy are illustrated in the PRISMA-ScR selection process flow diagram in Figure 2.

After conducting the initial search, duplicate articles were removed manually using Microsoft Office Excel. Two independent authors analysed titles and abstracts for their relevance and fulfilment of eligibility criteria. Subsequently, two authors reviewed the full texts of titles that appeared to meet the inclusion criteria or in which the abstracts did not provide sufficient information to make a decision. Discrepancies in screening of titles/abstracts and full-text articles were resolved through a discussion between the authors. A third author was consulted to provide a final decision in case of disagreement.

## 3 Results

The electronic search provided 598 records, including 78 from PubMed/Medline, 261 from Web of Science, and 259 from Scopus. After screening and duplicate removal, the full text of 87 were retrieved and reviewed for inclusion. Forty *in vitro* studies were considered eligible. The studies evaluated 3 mol% yttria-stabilized zirconia polycrystals (3Y-TZP) (29 studies), 4/5 mol% yttria-partially stabilized zirconia (4/5Y-PSZ) (6 studies), alumina (2 studies), alumina-zirconia composites (3 studies), lithium disilicate (3 studies), fluorapatite glass-ceramics (1 study), and porcelain (1 study). Vat polymerization was the most used technique, including 21 studies of digital light processing (DLP) and 15 studies using stereolithography (SLA). The only difference between DLP and SLA is the applied light source for photopolymerization: DLP cures a whole layer through a light mask generated by a digital micromirror device, while SLA solidifies each layer using an ultraviolet laser scanner from points to lines and areas. Additionally, material extrusion (ME) and material jetting (MJ) were also reported in 3 and 1 studies, respectively.

**Figure 3:**
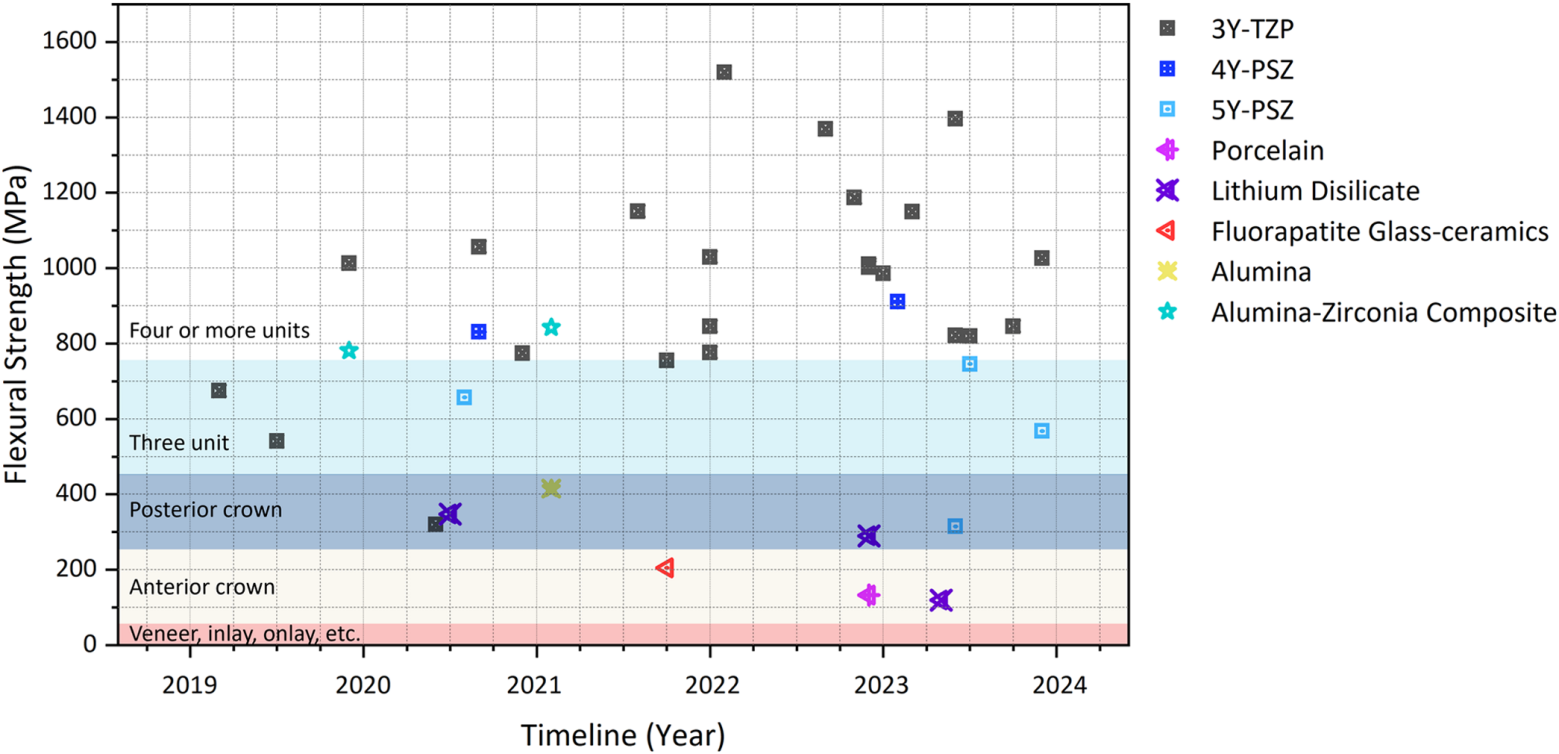
Timeline of reported flexural strength regarding different types of restorative ceramics. The different background colors refer to corresponding clinical indications as defined in ISO 6,872.

Table 1 presents values of density (15 studies), flexural strength (31 studies), fracture toughness (7 studies), Young's modulus (7 studies), hardness (11 studies), and other detailed information. Among these properties, flexural strength received the most concern due to its significance for the determination of indications according to the dental ceramic standard (15). Most studies adopted three-point bending tests; however, a wide strength variation was found for each ceramic. A timeline was plotted (Figure 3) to visualize the relation between publication/online date and reported flexural strength. Overall, the strength values (except for lithium disilicate) exhibit an upward trend, demonstrating the development of AM in the last 5 years. Additionally, only 7 records investigated the mechanical performance, such as bond strength to porcelain [2 studies (27, 54]), aging [2 studies (24, 31]), fatigue [2 studies (55, 56]) and wear [1 study (57]). Typical processing defects in AM ceramics were also addressed, including porosity, agglomerate, cracks, surface defects and other defects (large particles, deformation, contamination, manual defects, etc).

**Table 1:** Summary of the included studies and mechanical properties of dental ceramics produced by AM.

Reference	Ceramic	Technique	Other variables	Density (g/cm3) and/or relative density (%)	Flexural strength (MPa)	Fracture toughness* (MPa√m)	Young's modulus (GPa)	Vickers hardness (HV)
Jang et al. (17)	3Y-TZP	DLP	Volume fraction 48 vol%		94.25			
			Volume fraction 58 vol%		674.7			
Lian et al. (18)		SLA		6.026 (99.3%)	541 ± 160			
Borlaf et al. (19)		DLP		5.98 ± 0.02 (98.8%)	775			
Lu et al. (20)		DLP	3-point bending test	6.02 ± 0.02	1012.7 ± 125.5 737.4 ± 99.5			
			Ring-on-ring test					
Su et al. (21)		SLA	Pristine; layer thickness 20 μm	99%	1,057 ± 98		186 ± 15	1,337 ± 18
			Pristine; layer thickness 40 μm		874 ± 136		168 ± 18	1,306 ± 23
			Recycled; layer thickness 40 μm		389 ± 24		119 ± 25	1,300 ± 34
Branco et al. (22)		ME		5.9 ± 0.1				
Revilla-Leon et al. (23)		SLA			320.32 ± 40.55			
Zhai et al. (24)		SLA			776.7 ± 77.0			
		DLP			845.6 ± 183.5			
Xiang et al. (25)		SLA	0° layer orientation; polished		1151.1	12.635^a^		
			0° layer orientation; unpolished		967.0			
			90° layer orientation, polished		225.44	9.276^a^		
			90° layer orientation, unpolished		206.73			
Abualsaud et al. (26)		SLA	0° layer orientation	5.978	1186.73 ± 283.47			1609.54 ± 87.55
			45° layer orientation	5.942	810.92 ± 148.84			1634.96 ± 98.1
			90° layer orientation	5.987	521.51 ± 88.76			1676.61 ± 37.77
Baysal et al. (27)		MJ			1030.0 ± 29.2			1169.2 ± 48.4
Mei et al. (28)		DLP		6.02 (99.0%)		3.43 ± 0.29^b^	221.4 ± 2.2	1,189–1,193
Miura et al. (29)		SLA	0° layer orientation		1003.37	5.04^b^	173.33 ± 4.51	1300.30
			45° layer orientation		847.80	4.99^b^	179.67 ± 2.31	1257.78
			90° layer orientation		497.11	5.19^b^	187.33 ± 2.52	1311.16
Revilla-Leon et al. (30)		DLP			1,519 ± 254			
Tan et al. (31)		DLP	High-speed sintering	6.020 (99.26%)		5.75^b^		
			Conventional sintering	6.027 (99.44%)		6.83^b^		
Zandinejad et al. (32)		SLA			755.1 ± 147.1			
Zenthofer et al. (33)		DLP	No color infiltration		1,369			
			Intermediate color infiltration		1,197			
Han et al. (34)		DLP	No plasticizer		302			
			20% plasticizer		1,150			
Wang et al. (35)		SLA			820 ± 59			
Wang et al. (36)		SLA	Undoped		985.53 ± 94.73			
			0.14 wt% Fe2O3:		879.70 ± 77.10			
Lu et al. (37)		SLA	Parallel layer orientation	6.004 (98.75%)	1396.43 ± 230.08			
			Perpendicular layer orientation		1057.38 ± 203.60			
Giugliano et al. (38)		DLP			845.75 ± 266.16			
Jiaxiao et al. (39)		MJ		99.3%	1,010			1,621**
Mirt et al. (40)		DLP			1,027 ± 236			
Teegen et al. (41)		ME	1,350℃	5.89 ± 0.19	618 ± 131			
			1,450℃	6.01 ± 0.16	766 ± 123			
			1,550℃	6.05 ± 0.25	822 ± 174			
Kim et al. (42)	4Y-PSZ	DLP		99.4%	831 ± 74			
Yang et al. (43)		DLP			911			
Marsico et al. (44)	5Y-PSZ	DLP	0° layer orientation		657 ± 84		215 ± 1	1,328
			45° layer orientation		296 ± 11		209 ± 1	1,326
			90° layer orientation		126 ± 18		191 ± 5	1,327
Mirt et al. (40)		DLP			568 ± 128			
Wang et al. (45)		SLA	40 vol% solid loading	5.87 ± 0.02	702 ± 75			1,285**
			44 vol% solid loading	5.92 ± 0.03	723 ± 74			1,285**
			48 vol% solid loading	5.98 ± 0.02	735 ± 81			1,295**
			52 vol% solid loading	6.01 ± 0.02	746 ± 75			1,295**
Teegen et al. (41)		ME	Sintering temperature 1,350℃	5.37 ± 0.18	143 ± 9			
			Sintering temperature 1,450℃	5.70 ± 0.14	135 ± 9			
			Sintering temperature 1,550℃	5.63 ± 0.08	315 ± 38			
Wang et al. (46)	Porcelain	DLP			132.58 ± 25.83			
Baumgartner et al. (47)	Lithium disilicate	DLP	As fired; 25 μm layer thickness	2.508	346.3			
			As fired; 50 μm layer thickness		263.4			
			Polished; 25 μm layer thickness		431.3			
			Polished; 50 μm layer thickness		347.9			
			Glazed; 25 μm layer thickness		353.4			
			Glazed; 50 μm layer thickness		328.2			
Marsico et al. (48)		DLP	0° layer orientation		290 ± 60	2.01^c^	168 ± 3	
			45° layer orientation		75 ± 42	2.13^c^	148 ± 9	
			90° layer orientation		130 ± 84		165 ± 7	
Abreu et al. (49)		ME			120 ± 33.9			
Yang et al. (50)	Fluorapatite glass-ceramics	SLA			205.97		97.06	
Uçar et al. (51)	Alumina	DLP				6.5 ± 1.5^d^		
Coppola et al. (52)		DLP		3.92 (98.5%)	415		334 ± 16	
Wu et al. (53)	Alumina-toughened zirconia	SLA		98.11%		6.42 ± 0.33^b^		
Borlaf et al. (19)		DLP		5.37 ± 0.04 (98.5%)	781			
Coppola et al. (52)	Zirconia-toughened alumina	DLP	15 vol% ZrO_2_	98.5%	693 ± 87		318 ± 15	
			50 vol% ZrO_2_	98.8%	843 ± 67		268 ± 4	
			85 vol% ZrO_2_	99.2%	764 ± 136		213 ± 15	

* The test method for fracture toughness in included studies: ^a^. Single-edge V-Notch Beam; ^b^. Indentation fracture; ^c^. Chevron notch; ^d^. Fractographic analysis.

** The hardness value was transferred from GPa accordingly.

## 4 Discussion

### 4.1 Mechanical properties of AM ceramics

#### 4.1.1 Polycrystalline ceramics

3Y-TZP stands out as the strongest ceramic material for heavy load-bearing areas where aesthetics is not a primary concern (58). 3Y-TZP blocks have a density of 6.04–6.07 g/cm^3^ (relative density >99%), exhibiting excellent mechanical properties with the highest flexural strength among available ceramics, fracture toughness of 3.3–7 MPa√m, Young's modulus of 200–220 GPa, and Vickers hardness around 1,300 HV (20, 44, 58–61). Regarding AM 3Y-TZP, density ranges from 5.90 to 6.03 g/cm^3^ (98.8% < relative density < 99.4%) (18–22, 26, 28, 31). No difference in density (20, 26, 28, 31) was observed between AM and SM zirconia, while one study showed higher density for milled zirconia (22). Higher density was justified due to the characteristics of the raw material, such as purity and granulometric distribution of powder particles. The flexural strength of AM 3Y-TZP ranged from 320 to 1,519 MPa (17–20, 23, 24, 26, 27, 29, 30, 32, 33), which can be attributed to the different processing parameters during slurry preparation, printing, and post-treatments. While for milled, it ranges from 915 to 1,507 MPa (20, 23, 24, 26, 27, 30, 33). Six studies reported 6 to 65% lower flexural strength for AM samples (20, 23, 24, 26, 27, 33) compared to SM. This could be attributed to differences in microstructure and higher variation in defect type and size. On the contrary, two studies showed higher flexural strength for the printed ones, being 34 to 55% higher than the milled specimens (30, 32). Most of the researchers demonstrated higher Weibull modulus for milled specimens (20, 23, 29, 32, 33), except for only one study (26), suggesting higher reliability of conventional milling. Regarding other mechanical properties, two studies compared the fracture toughness of AM and SM 3Y-TZP, indicating no difference between them (28, 31). Among 3 studies, 1 reported similar Vickers hardness for both techniques (26), while the others found higher hardness for SM (27, 28), maybe due AM samples' porosity. One study showed similar Young's modulus (220 GPa) (28).

The limited translucency of 3Y-TZP has prompted the development of the third-generation zirconia. By increasing the yttria content to 4–5 mol% to incorporate more cubic phase, translucency is improved while the strength is compromised (42, 58, 62). 4Y-PSZ blocks offer a flexural strength of 748–952 MPa and a fracture toughness ranging from 2.50 to 3.50 MPa√m, making it a viable alternative to 3Y-TZP in aesthetic applications (63–65). 5Y-PSZ blocks have flexural strength of 557–681 MPa and fracture toughness ranging from 2.20 to 2.70 MPa√m (66, 67). In addition, milled 4Y- and 5Y-PSZ exhibit Vickers hardness (1,300 HV), density (6.00 g/cm^3^) (64, 68, 69) and Young's modulus (200–210 GPa) comparable to 3Y-TZP. However, few literature addresses AM 4/5Y-PSZ. Two studies produced 4Y-PSZ through DLP and achieved flexural strength of 831 MPa (42) and 911 MPa (43), both within the range of SM 4Y-TZP. The flexural strength of AM 5Y-PSZ varied (315–746 MPa) according tothe printing technique (40, 41, 44, 45), while hardness (1,285–1,328 HV) (44, 45) and Young's modulus (191–215 GPa) (44), were comparable to SM. A significant effect of building orientation was observed on the flexural strength and Young's modulus of 5Y-PSZ by DLP (44). 0° (parallel to the building platform) generated the highest values, while the weakest group of 90° reduced the strength in 80% and the Young's modulus in 11%; which was attributed to the layer line-associated defect. Hardness was not influenced by building orientation. Teegen et al. (41) used robotic material extrusion and observed a benefit of sintering temperature on grain size and flexural strength. For SLA, Wang et al. (45) found lower flexural strength at lower solid loading, despite no significant difference in density, grain size and hardness.

In dentistry, alumina using CAD/CAM has been employed as a core material for crowns and anterior 3-unit FPDs for more than 2 decades (5, 7). In the present review, two studies investigated the mechanical properties of 3D-printed alumina. However, it is important to note that these studies did not directly compare 3D-printed alumina to milled alumina. Coppola et al. (52) found that printed alumina exhibited lower flexural strength (415 MPa) and Young's modulus (334 GPa) compared to milled alumina. The printed alumina's density was 3.92 g/cm^3^ (98.5% relative density) with a Vickers hardness of 1,973 HV, exceeding milled alumina's range. In contrast, Ucar et al. (51), reported a higher flexural strength (490 MPa) and fracture toughness (6.5 MPa√m) for alumina, while the printed alumina had slightly lower Vickers hardness (1,581 HV) than milled alumina. While alumina still obtains attention in the research related to implants and abutments, its application for dental restorations has decreased in recent years. The reason for this is that mechanical properties like flexural strength and translucency are inferior compared to other ceramics. Moreover, the high hardness of alumina is another limitation against machining these ceramics. The stiffness of alumina is about 10 times higher than that of dentine, which restricts its application in situations where achieving a high level of elastic compatibility between the tooth structure and the prosthesis is necessary (7).

Alternative to the previous materials, alumina-zirconia composites have gained significant attention in the field of dentistry because of their unique properties. These reinforced composites can be categorized based on the zirconia content, known as zirconia-toughened alumina composites when containing a relatively low amount of ZrO_2_ (5–20 wt%) for enhanced toughness and reliability, or alumina-toughened zirconia, where larger alumina particles embedded in a fine zirconia matrix provide increased toughness, hardness, and strength. It is essential to attain fully dense and finely structured microstructures in these composites with an even distribution of the two phases within the material to achieve high mechanical properties (52). In this review, three studies examined the mechanical properties of various AM alumina-zirconia composites without comparison with CAD/CAM composites. In one study (52) composites with different alumina contents (15, 50, and 85 vol% of ZrO_2_), the composite with 15 vol% ZrO_2_ demonstrated the most favorable combination of high hardness (2,156 HV) and flexural strength (693 MPa), with a Young's modulus of 318 GPa and a density of 4.20 g/cm^3^. Other study (53) investigated the fracture toughness and Vickers hardness of alumina-toughened zirconia and reported the highest values of 6.4 MPa√m and 1,290 HV respectively, at the highest alumina content (3.9 wt%). Lastly, Borlaf et al. (19) developed slurries of alumina-toughened zirconia, containing 20 wt% alumina, using either a one-step or a two-step procedure. The two-step method led to delamination problems, resulting in lower values of flexural strength (222–285 MPa) and Weibull modulus (3.07). However, the one-step procedure exhibited a flexural strength of 781 MPa and a Weibull modulus of 10.48, without delamination problems.

#### 4.1.2 Glass-ceramics

Traditional feldspathic porcelain stands out due to its exceptional aesthetic characteristics, while it is the weakest among dental ceramics. Milled feldspathic has been utilized for many years as one of the oldest block materials owing to its satisfactory translucency (58, 70). In an attempt to improve their strength, dental companies have used leucite as a reinforcement within the material matrix. Leucite-reinforced ceramics exhibit excellent optical characteristics, whereas the improvement of strength was minimal (70). As a result, these ceramics have limited indication since they may not provide sufficient strength and durability. Instead, they are commonly utilized as veneers on minimally prepared anterior teeth, where the focus is primarily on enhancing aesthetics (58). AM offers an advantage over milling in the production of thin veneers by avoiding edge damage (46, 71). AM feldspathic veneers presented an average flexural strength of 133 MPa (46). Although the strength is similar to the reported values for SM (135 MPa) (58), it revealed a low Weibull modulus of 3.93, indicating that reliability is yet to be improved.

Lithium disilicate, known as one variant of dental lithia silicate glass–ceramics, exhibits a unique microstructure characterized by interlocking needle-like crystals embedded within a glass matrix. This particular morphology redirects crack propagation around each individual lithium disilicate crystal, resulting in increased strength and toughness (2, 72). Due to its excellent mechanical properties, particularly in terms of aesthetics, this ceramic material is preferred for creating veneers, inlays/onlays, and single crowns. Lithium disilicate blocks are widely used in dental lab or chairside, and can undergo wet milling in pre-crystallized phase (e.g., IPS e.max® CAD, Ivoclar) or fully-crystallized phase (e.g., Initial™ LiSi Block, GC). Lithium disilicate ceramics usually exhibit density of 2.4–2.6 g/cm^3^, flexural strength of 200–500 MPa, fracture toughness of 1.3–2.2 MPa√m, Young's modulus of 90–110 GPa, and hardness of 6–8 GPa, after crystallization (2, 3, 5). The reported flexural strength of AM lithium disilicate varies considerably from 120 MPa to 431 MPa, which could be attributed to limited number of studies and different AM techniques. Baumgartner et al. (47) produced lithium disilicate via DLP with different finishing protocols and layer thicknesses, which had densities of around 2.5 g/cm^3^. The strongest group was achieved through polishing and a thinner printing layer thickness of 25 μm (431.3 MPa), which was within the reported values for milled lithium disilicate. In addition, the lower Weibull modulus was observed in the polished samples, possibly due to the inconsistent distribution of surface flaws generated during polishing. The “as fired” and glazed specimens exhibited higher Weibull modulus, indicating high surface quality. Other study (48) observed higher flexural strength of 290 MPa when using 0° orientation for building directions but no difference for hardness (5.5 GPa), which is slightly below the reported values for milled lithium disilicate. Young's modulus was found to be 168 GPa for the 0° group and 165 GPa for the 90° group, with the 45° printing orientation having a significantly lower value of 148 GPa. Moreover, fracture toughness was determined to be similar or higher to the reported values for milled lithium disilicate. These findings highlight the potential of DLP as a viable alternative with favorable mechanical properties. Abreu et al. (49) adopted the RC technique to fabricate lithium disilicate, while the flexural strength and hardness were only 37% and 72% of that by SM.

Fluorapatite glass-ceramics have also attracted considerable attention due to impressive biocompatibility, aesthetic properties, and good mechanical characteristics. Their composition includes a glass phase and a needle-like crystal phase known as fluorapatite (FAp), which resembles the crystal structure of enamel (73). This unique combination allows for the release of trace amounts of fluorine and exhibits a similar morphology to natural enamel, which promotes excellent biocompatibility and enhanced resistance to acid (74). A study investigated the mechanical properties of AM fluorapatite, and observed higher flexural strength, Vickers hardness and elastic modulus (respectively, 205.97 MPa, 772.05 HV and 97.06 GPa) than dry-pressed specimens (160 MPa, 660 HV and 94.8 GPa) (71). This suggests AM as an alternative also for fluorapatite. Nevertheless, in clinical settings, fluorapatite face certain challenges related to their mechanical and tribological properties, which can lead to excessive wear and fractures, which restricts their range of applications (75).

### 4.2 Processing defects

Owing to the brittle nature, the mechanical properties of ceramics are highly sensitive to processing defects. As the currently available AM techniques combine both 3D printing and conventional manufacturing, such as debinding and sintering, which can result in defects by both manufacturing techniques. In this review, a variety of defects were found in the final products as: porosity, agglomerates, cracks, surface defects, large particles, delamination, deformation, etc. While some of these defect types are also common in conventional techniques, there can be differences in shapes, sizes, and locations due to different formation causes, resulting in different influences on the mechanical properties of the final products.

Porosity, as a common type of defect for both AM and SM, can be formed during the entire AM process, including slurry preparation, printing, debinding, and sintering, existing as voids or bubbles in the final products. Highly viscous ceramic slurry can trap air bubbles during preparation or printing, which are not effectively removed by subsequent thermal treatments, leading to micro-sized residual pores of diverse shapes in the final parts (20, 22, 26, 76, 77). Additionally, various situations can contribute to the formation of porosity or cracks: weak bonding between successive layers can lead to insufficient layer fusion (23, 25); solvent evaporation from the ceramic paste as it dries on the exposed surface before the next layer is added may cause uneven shrinkage during sintering (76); sedimentation of ceramic particles can lead to voids between layers after sintering (78, 79). For post-treatments, the removal of binders during the debinding process can also leading to the presence of residual pores in ceramic parts (20, 76). Unsuitable sintering conditions (52) or insufficient ceramic particles dispersion (76) may not be fully densified, resulting in the formation of pores. Porosity have been addressing as one main type of critical defects in AM ceramics. Compared with milling, it was found that DLP zirconia have a larger scale of critical defect size, which can explain its relatively low Weibull modulus (20). Despite zirconia, Abreu et al. (49) also found individual surface pores as the fracture origin of lithium disilicate by robotic material extrusion, while Marsico et al. (48) observed porous region as one of the main fracture initiations in lithium disilicate by DLP. The existence of porosity could also affect hardness, resulting in lower value in comparison with milled ceramic with similar compositions (27, 28).

Agglomerate is another common defect for AM and SM. Agglomerates are clumps or clusters of particles that stick together in a material, which can be attributed to incomplete dispersion during the slurry-forming process (44). The high viscosity of ceramic slurry can hinder the uniform dispersion of these particles (21). Furthermore, the presence of residual powder particles or incomplete removal of powder can lead to the creation of agglomerates in the printed ceramic structure (20). Additives or binders in the ceramic formulation may also influence particle interactions and lead to agglomeration (80). During sintering, agglomerations can persist within the ceramic parts (81). The presence of agglomerates can act as stress concentrators, leading to the initiation of crack propagation and fractures, consequently reducing the overall strength of the material (82). In addition, they correspond to large under-densified zones, which can have negative effects on its translucency and mechanical properties (25).

Cracks, including microcracks along the interface of layers and macrocracks, pose a significant risk to the material strength in AM ceramic. These cracks mainly initiate and propagate at the layer interfaces and grain boundaries, where the bonding is weaker compared to other regions (82). High solids volume fraction is essential to achieve sufficient strength. In the study by Jang et al. (17) and a decreasing zirconia volume fraction resulted in an increasing number of cracks. This can be attributed to a low solid volume fraction, which results in the presence of undensified regions, thereby leading to porous regions along the layers. While maintaining a high solid volume fraction is important, the ceramic slurry needs to retain sufficient flowability and dispersibility to achieve a high quality of the final product (17). Transportation, handling, and movement of the green part throughout the printing and post-processing stages may lead to deformation. Consequently, the green part can generate cracks along the layers' interface due to the influence of uneven internal stress (82). Additionally, uneven shrinkage during the debinding and sintering processes can induce stress at the interfaces between different layers, resulting in the formation of cracks and deformation of the green part (83). Moreover, a reduced solid content in the ceramic slurry results in a higher proportion of polymer content in the green part, which leads to increased shrinkage during curing, consequently leading to internal stress (76). In addition, particle segregation and sedimentation of coarse particles after sintering can lead to larger particles at layer boundaries, which can lead to inhomogeneous grain distribution after sintering and different mechanical properties in different printing layer orientations. This can lead to the formation of cracks and compromise the mechanical properties (79). Delamination was observed as a result from weak bonding strength between successive layers (25). This can cause weak interfaces, making the green part more vulnerable to cracks. In addition, thicker layers can lead to layer union issues because larger layer line defects or even delamination of the layers can occur (44, 84).

Another concern for AM ceramics is surface roughness, or surface defects, as high roughness has been proven to impact mechanical properties, fit, aesthetics, bacterial adhesion and wear on opposing teeth or restorations (26, 67, 85). The main contributor to roughness is the “Step effect” or visible layer lines caused by layer-by-layer construction (21, 44), while the residual ceramic slurry after printing serves as the secondary factor (37). A study on SLA zirconia (37) showed that the surface parallel to the printing platform exhibited an average surface roughness of 0.71 μm (Ra), while the surface perpendicular to the printing platform was significantly rougher (2.91 μm). Both orientations considerably exceeded the required value (0.2 μm) for dental restorations (86), indicating the significance of subsequent surface finishing, such as polishing or glazing. Thorough surface polishing has been proven to reduce the effect of roughness, therefore significantly promoting the flexural strength and fatigue strength of AM zirconia (25, 37, 55). Conventional glazing technique or glass infiltration can also generate a shiny and smooth surface for monolithic ceramics, while their influence on mechanical properties of AM ceramics yet lacks reports.

Other microscopic and macroscopic defects, such as large particles, deformation, contamination, and machining damage, were also observed previously (20, 29, 53, 82). Previous studies also reported small amounts of heavy metals detected in zirconia, possibly originating from contamination during the purification and production processes (29, 87). Furthermore, printed zirconia is very sensitive to manual defects introduced before sintering and after printing because of the extreme fragility of the products (37).

### 4.3 Effect of printing layer orientations

Printing layer orientation has been recognized as a significant factor influencing the mechanical properties of AM materials. Studies have shown that AM ceramics can achieve the highest flexural strength, Young's modulus, and fracture toughness when the printing layers are perpendicular to load in the bending tests (25, 26, 29, 44). One explanation is that this orientation avoided the step effect on the tensile surface, thereby reducing the effect of surface roughness on flexural strength (44). Though the step effect can be removed by delicate surface polishing, sometimes microcracks along the layers still remain. When printing layers are perpendicular to the load, these interlayer cracks are less likely to appear in the zone where the stress is concentrated. The effect of orientations could also be related to the difference in microstructures of different orientations. During the printing process, large particles are likely to settle before the layer is cured, resulting in coarse grain at the bottom (79). Additionally, Lu et al. (37) observed the presence of elliptical-shaped pores that acted as fracture initiators, in the long axis parallel to the layers. These pores may be distributed within the sintered ceramic, but specimens with a printing orientation parallel to the load may be more prone to the influence of these pores due to their sharper shape in the direction. However, it is yet unclear how the different mechanical properties of different printing layer orientations would influence the mechanical performance of clinical restorations, and if this risk can be eliminated by optimization of restoration design and printing orientation/angle.

### 4.4 Clinical performance

**Figure 4:**
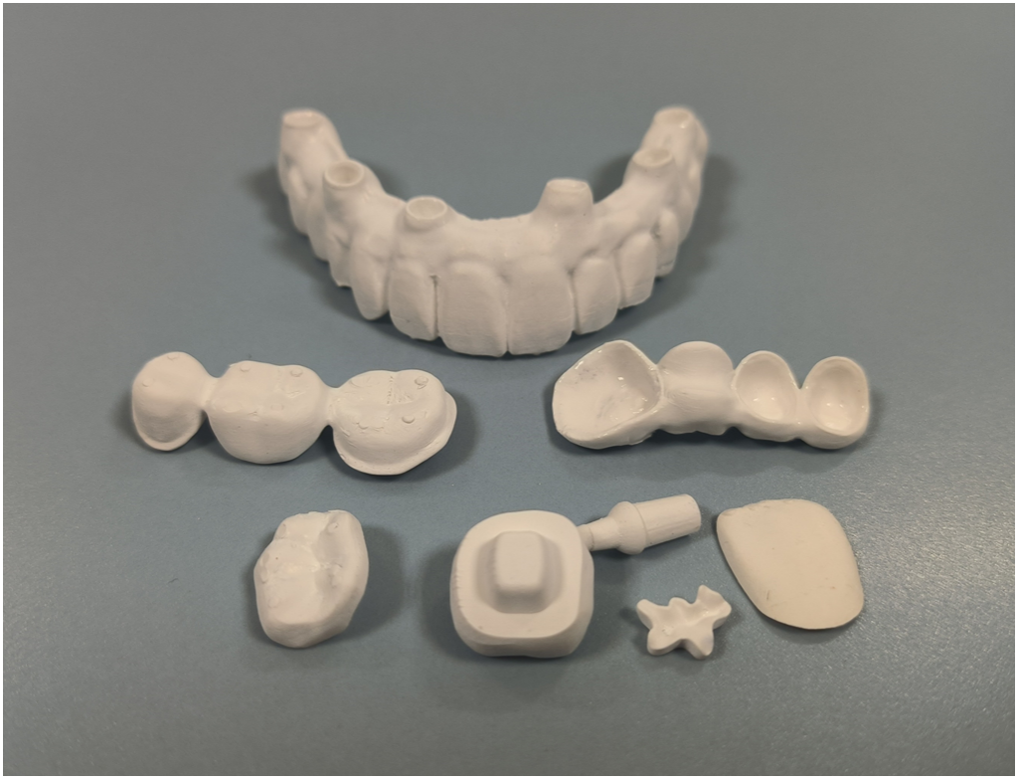
Various permanent dental ceramic restorations manufactured by SLA, including single crown, multi-unit restorations, veneer, inlay, onlay, post and core, endocrown, implant supratructure and abutment.

Ceramic restorations fabricated by AM can exhibit a satisfying visual quality, including structural integrity, smooth surface, and fine details, indicating the promising clinical application of the technique, as shown in Figure 4. However, literature regarding the clinical performances of AM restorations is still very limited. The fracture resistance of AM 3Y-TZP crowns was reported as weaker than SM crowns both before and after 1.2 million loading cycles (56). While another study found that AM 3Y-TZP can achieve a comparable fatigue strength to SM 3Y-TZP by optimizing printing layer orientation and surface polishing (55). Regarding low-temperature degradation on AM 3Y-TZP, an increase in the monoclinic phase can be observed, without a significant decreasing in flexural strength (24). However, hardness and fracture toughness (31) seem to be affected by aging. For wear, both DLP-manufactured 3Y-TZP specimens with horizontal and vertical printing orientations have similar friction efficiency and negligible volumetric substance loss after occlusal wear by zirconia antagonists (57). In addition, no difference was found between the bond strengths of printed and milled 3Y-TZP substrate and porcelain veneer, by either Schwickerath adhesion test (54) or shear bond strength test (27). However, the adhesion between AM ceramic and abutment tooth is another essential topic, but relevant reports are still lacking. Beyond geometry, clinical performance can be compromised by defects that directly impact the ceramic's structural integrity and longevity. Layer cracks and delamination are common in additively manufactured ceramics due to insufficient bonding between layers and accumulated internal stresses. These cracks can act as initiation points for failure, particularly in the cyclic loading environment of the oral cavity where chewing forces can exacerbate structural weaknesses. Furthermore, density variations and porosity arise from incomplete filling between layers or air entrapment during printing, leading to stress concentration points that reduce the overall mechanical robustness. Such inconsistencies are especially concerning for dental restorations, as they may lead to premature wear or fracture under masticatory loads (82).

### 4.5 Future prospect

In this review, AM ceramics have reached a level close to milled ceramics in terms of mechanical properties, but the reliability of the final product is still a general problem associated with processing defects. Therefore, technological development and further research are still necessary for defect identification and control to improve the reliability of the materials and fabricated restorations. The second common issue of AM ceramics is the influence of printing layer orientations. Though some studies have addressed the strongest or weakest orientations for standard specimens, the situation of clinical prostheses is much more complex. Yet it is unclear how the real restorations would be impacted and if/to which extent such effects can be avoided by the optimization of design. Regarding different ceramics, while 3Y-TZP has received the most in-depth investigations, its long-term clinical performance as a framework material requires further research, such as the duration of the restorations and the interaction with the bonding to natural teeth or porcelain veneer. For monolithic zirconia and glass ceramics, scientific reports are still very limited in comparison to 3Y-TZP. More technological explorations that combine both mechanical and optical properties are expected for these materials. Additionally, the effects of humidity and wear in the oral medium are of concern when evaluating the properties. Literature investigating surface treatments, coatings, or post-processing methodologies to improve surface quality and toughness are still lacking from the literature and should be encouraged. The future trends for the development of dental ceramic 3D printing include the development of higher-performance and more reliable ceramics, translucent ceramics for aesthetic applications, biomimetic gradient ceramics through multi-material printing, and advanced technologies to increase production efficiency.

## 5 Conclusion

With significant technological advancements, AM ceramics come close to milled ceramics in terms of mechanical properties; however, they are still considered to be inferior in terms of reliability and influence of printing layer orientations, which can be attributed to the higher variety of processing defects. 3Y-TZP is the most developed AM ceramic, whereas scientific documents regarding long-term clinical performance are required for its clinical applications. Further exploration is still needed for the fabrication of translucent ceramics such as monolithic zirconia and glass ceramics.

## Data Availability

The original contributions presented in the study are included in the article/Supplementary Material, further inquiries can be directed to the corresponding author.
